# Knowledge about HPV and Screening of Cervical Cancer among Women from the Metropolitan Region of Natal, Brazil

**DOI:** 10.1155/2013/930479

**Published:** 2013-03-31

**Authors:** Érika Galvão Lima, Diego Breno Soares de Lima, Cleine Aglacy Nunes Miranda, Valeska Santana de Sena Pereira, Jenner Chrystian Veríssimo de Azevedo, Josélio Maria Galvão de Araújo, Thales Allyrio Araújo de Medeiros Fernandes, Paulo Roberto Medeiros de Azevedo, José Veríssimo Fernandes

**Affiliations:** ^1^Post-Graduate Program in Biological Sciences, Biosciences Center, Federal University of Rio Grande do Norte, Avenida Sen. Salgado Filho s/n, Campus Universitário, Lagoa Nova, 59072-970 Natal, RN, Brazil; ^2^Pediatric Hospital, Federal University of Rio Grande do Norte, Rua Gal. Cordeiro de Farias, s/n, Petrópolis, 59012-570 Natal, RN, Brazil; ^3^Department of Microbiology and Parasitology, Federal University of Rio Grande do Norte, Avenida Sen. Salgado Filho s/n, Campus Universitário, Lagoa Nova, 59072-970 Natal, RN, Brazil; ^4^Department of Biomedical Sciences, University of Rio Grande do Norte State, Rua Atirador Miguel Antônio da Silva Neto, s/n, Aeroporto, Mossoró, RN, Brazil; ^5^Department of Statistics, Center of Exact Sciences and Earth, Federal University of Rio Grande do Norte, Avenida Sen. Salgado Filho s/n, Campus Universitário, Lagoa Nova, 59078-970 Natal, RN, Brazil

## Abstract

*Objective*. The purpose of this study was to assess the knowledge level about HPV and screening of cervical cancer in women from the metropolitan region of Natal, Brazil. *Materials and Methods*. A descriptive cross-sectional study involving sexually active women was conducted. The participants were submitted to a face-to-face interview, using a structured questionnaire that permitted the quantification of data and opinions of the respondents. *Results*. Most participants (70.9%) had poor knowledge about HPV and also the Pap test (53.0%). The high level of knowledge about HPV was associated with age, education, marital status, household income, and pregnancy, while the high level of knowledge about the Pap test proved to be associated only with education and household income. *Conclusion*. The results highlight the need for performing educational campaigns emphasizing the role of HPV in the etiology of cervical lesions of different degrees, including cervical cancer, as well as the importance of having a Pap test regularly to prevent these diseases.

## 1. Introduction

Cervical cancer is the third most common cancer affecting women, after nonmelanoma skin cancer and breast cancer, and is the third leading cause of death by cancer among women worldwide, with higher incidence in developing countries [1, 2]. It is estimated that about 500,000 new cases are reported every year, with approximately 230,000 deaths worldwide. In Brazil, the crude incidence rates per 100,000 women, estimated for the year 2012, were 17 for the country and 14 for the Rio Grande do Norte State. The incidence of the disease starts from the age of 20 and the risk gradually increases with age, reaching its peak generally at age 50 to 60 [3].

The natural history of cervical cancer reveals that, despite its high incidence, this malignant neoplasm stands out among those with the greatest potential for prevention and cure in view of their infectious nature [4]. Cervical cytology is one of the most efficient methods for the screening of cervical cancer and is the most commonly used method worldwide [5]. Despite its benefits, many women in developing countries have never been screened or are not screened at regular intervals, so that this screening program does not have the desired impact on women's health [6]. Reasons for the lack of effectiveness of these screening programs in developing countries include low screening coverage and participation rates among women, lack of quality control, poor ability of the health care system to offer proper followup and access to colposcopy, and inappropriate final diagnosis and treatment [7]. 

There is no doubt that when women face participation decisions, it is ideal for them to have a good understanding and perception of the risk of acquiring cervical cancer and the benefits of preventing it [7]. In Brazil, the coverage of the cytopathology exam has not still reached the desired indices. It is estimated that approximately 40% of Brazilian women have never undergone the procedure [3]. This is due to several factors, including the difficulty of access to health services, poor knowledge about the Pap smear, and the lack of awareness of the benefits that this exam brings to women's health [8]. 

Human papillomavirus (HPV) is one of the most common causes of sexually transmitted disease in both men and women around the world, especially in developing countries. The prevalence of asymptomatic infection varies from 2 to 44%, depending on the population and studied region [9]. Most HPV infections are transient and some studies show that the majority of sexually active individuals are exposed to and acquire infection from this virus at some phase in their lives.

HPV infection is more prevalent in young adults, at the beginning of their sexual activity, with a subsequent decline in the prevalence rate with increasing age, likely as a result of the development of an immune response against the virus and reduction of sexual activity [10, 11]. Currently, it is known with certainty that HPV is the etiologic agent of virtually all cases of cervical cancer and is responsible for a high proportion of preinvasive cervical lesions as well as genital warts and other nongenital cancers [12].

Today, more than 150 different HPV types have been cataloged and about 40 can infect the epithelial lining of the anogenital tract and other mucosal areas of the human body. Based on their association with cervical cancer and precursor lesions, HPVs are classified as high-risk (HR-HPV) and low-risk (LR-HPV) oncogenic types. LR-HPV types, such as HPV 6 and 11, can cause common genital warts or benign hyperproliferative lesions with very limited tendency to malignant progression, while infection with HR-HPV types, highlighting HPV 16 and 18, is associated with the occurrence of premalignant and malignant cervical lesions [13]. HR-HPV types are also associated with many penile, vulvar, anal, and head and neck carcinomas, and contribute to over 40% of oral cancers [14]. 

The recognition of the central role of HPV in the etiology of cervical cancer has dramatically changed the vision of how to prevent this cancer. Among the strategies, introduction of HPV testing for primary screening or as an adjunct test and the introduction of HPV vaccines to prevent HPV infection are being evaluated in different settings around the world [15]. 

Indeed, these advances are available in many countries that have approved the introduction of the HPV vaccine. However, gaps in knowledge about the causal role of HPV in cervical cancer and the benefits of preventing HPV infection may hamper the successful introduction of the technologies. The greater the women's knowledge of HPV and its role in the development of cervical cancer, the greater will be the adherence to preventive measures. 

The objective of this study was to evaluate the knowledge level about HPV and the Pap smear screening among high school students and women treated in the health units of the metropolitan region of Natal, Brazil.

## 2. Materials and Methods

### 2.1. Study Design and Participants

This was a descriptive cross-sectional study carried out during the period of January 2009 to December 2011 involving sexually active women of the metropolitan region of Natal, capital of Rio Grande do Norte State, Northeastern Brazil. We chose basic health units and public schools, distributed throughout all the 10 municipalities that make up the metropolitan region of Natal, to participate in the research.

 The interviewers approached potential female participants among the high school students of the selected schools and among women attending basic health units during the study period. In the schools, some classes were randomly chosen and the students were invited by interviewers to participate in the study. In the health units, women who sought by spontaneous demand the screening test for cervical cancer were invited to participate in the study. After agreeing to participate in the study, the women aged 18 years old or older signed an informed consent before survey administration. Those under 18 years had their participation authorized by parents or guardians. A total of  706 participants, aged 14 to 59 years, were included in the study. The only inclusion criteria were to be of female gender, to have already initiated sexual activity, and to agree to participate in the research. 

This study was approved by the Research Ethics Committee of Federal University of Rio Grande do Norte.

### 2.2. Data Collection

All interviews were conducted face-to-face by trained enumerators using a paper structured questionnaire, containing 25 questions including 15 questions about age, ethnicity, marital status, education, household income, sexual activity, and reproductive health history. There were six questions regarding Pap smear knowledge, including such questions as (1) Have you previously heard about this exam? (2) What is the purpose of this exam? and (3) What is the frequency with which the exam must be done? Other questions concerned the level of awareness of the respondents on the advantages and benefits of the examination for the health of women. The remaining four questions were related to HPV knowledge, including questions about the potential consequences of HPV and the awareness of the signs and symptoms, and mode of transmission. All the Pap test and HPV knowledge questions were accompanied by multiple-choice answers, one of which was considered the correct answer to the question. The interviewers were instructed to recite all multiple-choice answers to the participants and then ask for the answer to each question.

### 2.3. Data Analysis

We defined the level of knowledge according to the number of correct responses. Knowledge about Pap screening was categorized as high, medium, or low based on the six Pap-related questions in the survey. A participant with a high level of knowledge answered at least five of the six Pap knowledge questions correctly; participants with a medium level of knowledge answered three or four of the six questions correctly, and participants with a low level of knowledge answered none or at most two of the questions correctly. Women who responded correctly to all four HPV-related questions were categorized as having a high level of knowledge on the virus, those who answered two or three questions correctly were categorized as having a medium level of knowledge, and the women who responded correctly to only one or none of the HPV-related questions were categorized as having a low knowledge level. 

The participant ethnicity was defined based on self-reports according to the criterion of the Instituto Brasileiro de Geografia e Estatística (IBGE), which classifies ethnicity into five categories: white, black, mulatto, Asian, and “native,” which were combined into a nonwhite category. We considered as having low income those participants who reported a household income of up to the minimum wage (678.00 reais/month), equivalent to US$ 320.00; as middle income, those with a household income between one and four times the minimum wage, and as high-income, women who reported a household income higher than four times the minimum wage. For the purposes of analysis, women with high Pap screening knowledge were compared against those with low and medium knowledge. Similarly, women with a high level of knowledge about HPV were compared with those who demonstrated a low level of knowledge on the virus.

### 2.4. Statistical Analyses

To verify the association between the variables studied and the knowledge level for HPV and Pap test, we used the odds ratio (OR) and its 95% confidence intervals (CIs), according to the univariate regression model, using the software SPSS, version 17.0, and PEPI. Statistical significance within each group was evaluated by Pearson's *χ* ^2^ test.  *P*  value ≤ 0.05 was considered statistically significant. 

## 3. Results

### 3.1. Profile of the Participants

The profile of the segment of the population studied is presented in Table 1. The age range of respondents was 14–59 years with a mean of 30.4 years and SD of ±11.5 years. 

Most (51.8%) of the studied population consisted of young women aged 14 to 28 years, 52.3% were of nonwhite ethnicity, and 64.2% were either married or living in a stable relationship with her partner. Regarding the level of education, 37.1% reported that they had only elementary school level or less, 52.4% were attending high school or had completed high school, and only 10.5% reported being enrolled in or having completed some type of education above high school. The vast majority (78.9%) of the women interviewed reported a low income (household income of up to R$ 678.00 reais/month), equivalent to US$ 320.00. Most of them (52.8%) had never had a pregnancy, and among those who had been pregnant, the majority (81.0%) had between one and three children.

### 3.2. Pap Test Knowledge

**Figure 1:**
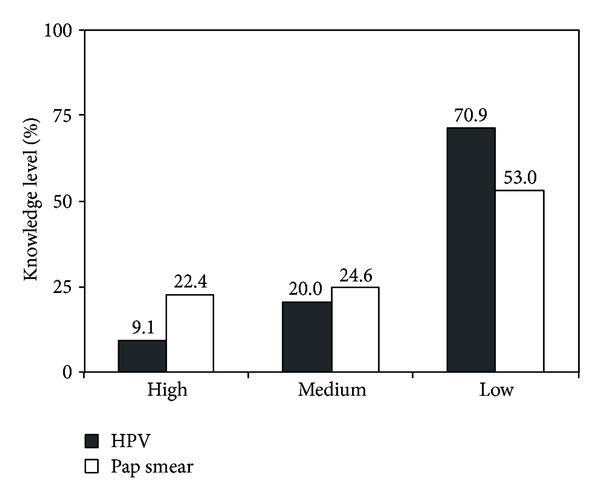
Distribution of knowledge level related to Pap test and HPV. A high proportion of the women had low knowledge related to both Pap test and HPV.

Participants of this study showed a low level of knowledge about Pap test screening. When asked if they knew what a Pap test was, 53.0% of the respondents indicated that they knew about the exam and were able to say what it means, and 48.0% of them were able to provide the correct frequency with which the test should be done and to demonstrate awareness of the advantages and benefits of performing the procedure regularly. According to the level of knowledge presented, participants were categorized as having low, medium, or high level of knowledge about the Pap test screening, based on the correct number of responses. Of the women surveyed, 22.4% exhibited a high level of knowledge about the Pap test, 24.6% had a medium knowledge level, and the majority (53.0%) were categorized as having a low level of knowledge about the exam (Figure 1). 

Table 2 shows the Pearson's Chi-square  *P*  values association between the considered variables and Pap knowledge. We found that the high knowledge level of the Pap test was associated with level of education (*P* < 0.001) and household income (*P* < 0.001), while the middle-level knowledge of this exam was associated with marital status (*P* < 0.001) and pregnancy (*P* < 0.001). Association between the Pap test knowledge level according to the considered variables and odds ratio (OR) can be seen in Table 4. 

Having or not having the high-level knowledge of the Pap test was considered the dependent variable, and independent variables were those identified as significant in Table 2. We found that having a high school education or above versus elementary school education or less (*P* < 0.001) and having a middle or high household income versus low household income (*P* < 0.001) were related to an increase of the Pap test knowledge score. The odds ratio of having a high Pap knowledge score was 10.2 times greater among women with high school or above, compared with those with elementary school or less (95% CI 5.534–8.825), and 2.68 times greater for the women with a middle or high household income, compared with those with a low household income (95% CI 1.810–3.987).

With regard to the level of knowledge of the Pap test, among the 706 women surveyed, 96.2% had heard about it, but only 47.0% had medium or high knowledge related to the procedure. The main source of information about the Pap test was health care staff, mentioned by 45.2% of the respondents, followed by friends and family with 22.5%. We found that 87.4% of the interviewed women reported that they had had at least one Pap test during their lifetime; 69.4% had had the procedure done with the frequency of at least once every three years, as recommended by the Ministry of Health; 18% said they had not had the test in the last 3 years, and 12.6% reported that they had never had the test throughout their life. When asked about the reasons for not having the procedure, the responses most commonly mentioned by these women were that they felt that nothing was wrong (28.6%), neglect (23.4%), had fear of pain (12.2%), and felt ashamed of exposing their intimacy (6.8%).

### 3.3. HPV Knowledge

Participants of this study presented a very low level of knowledge related to HPV. Only a minority of the women interviewed were able to quote correctly the transmission mode of the HPV and identify the signs and symptoms of the infection caused by this virus, as well as consciously recognize the relationship between the genital HPV infection and the occurrence of cervical lesions including cancer. A significant portion of respondents (64.2%) said they had heard about HPV, but a much smaller proportion (29.1%) demonstrated having some knowledge about the virus. Only 20.0% of the women respondents knew that HPV is a sexually transmitted agent, and a very small percentage (9.1%) of participants were able to establish the correlation between HPV and the occurrence of anogenital warts and of premalignant cervical lesions and cervical cancer. 

Thus, when subjects were analyzed according to the level of knowledge presented about HPV, it was found that most (70.9%) of the participants were categorized as having low knowledge about the virus; 20.0% exhibited a medium knowledge level, and only 9.1% were categorized as having a high level of knowledge about HPV (Figure 1).  Table 3 shows the analysis by means of Pearson's Chi-square to verify the existence of the association between knowledge of HPV and the variables studied.  *P*  values confirmed the existence of association between knowledge level of the virus with age, (*P* < 0.001), education (*P* < 0.001), household income (*P* < 0.001), pregnancy (*P* < 0.001), and marital status (*P* = 0.006).

Association between HPV knowledge level according to the considered variables and odds ratio (OR) can be seen in Table 5. Having a high knowledge level or not about HPV was considered the dependent variable, while independent variables were those identified as significant in Table 3. We found an increase of the HPV knowledge score in the women aged between 39 and 48 years (*P* < 0.001) and 49 to 59 years (*P* = 0.001) versus those aged between 14 and 28 years; in those having high school education or above versus elementary school education or less (*P* < 0.001); in the married or accompanied versus single (*P* = 0.003); and in those with middle or high household income versus low household income (*P* < 0.001). The odds ratio of having a high HPV knowledge score was 4.50 times greater in women aged 39–48 years (95% CI 2.289–8.859) and 3.71 times greater in those aged between 49 and 59 years (95% CI 1.718–8.001), compared with those aged between 14 and 28 years. Furthermore, we observed values of the odds ratio of 43.16 times greater in women with high school or above, compared with those with elementary school or less (95% CI 5.534–8.825); of 1.92 times greater in women married or accompanied, compared with single women (95% CI 1.056–3.507); 3.11 times greater in those with a middle or high household income compared to those with a low household income (95% CI 1.824–5.305); and 3.81 times greater in women who had at least one pregnancy, compared with those who had never been pregnant (95% CI 2.116–6.844).

## 4. Discussion

Cervical cancer screening is a diagnosis resource of fundamental importance to women's health in that it allows early detection of the precursor lesions and the therapeutic intervention, before they progress to the malignant form. Because of its infectious nature, cervical cancer has a high potential for prevention, which can be put into practice by avoiding risky behaviors for acquisition of the HPV infection, as well as through the use of available vaccines. Thus, knowledge about the role of HPV in the development of the disease becomes of paramount importance for improving the degree of awareness of women to adopt measures to reduce the risks of infection by this virus, as well as to have early diagnosis of the existing infections. In this study we evaluated the degree of knowledge about HPV and the Pap test in a segment of the female population of the metropolitan region in Natal, Brazil.

According to the official data, the metropolitan region of Natal consists of 10 municipalities including the capital, with a female population of approximately 367,768 women aged 14 to 59 years. This study focused only on the women in this age group, enrolled in public high schools, and the women being treated in basic health units in the municipalities involved, in the period from January 2009 to December 2011.

Our study showed that 87% of the surveyed women reported having had a preventive Pap test at least once in their lifetime and 69.4% said they had had the procedure according to the recommended frequency suggested by the Ministry of Health of Brazil. This coverage rate of the Pap test is similar to that found by Fernandes et al. [16] in a study involving women from a municipality in the metropolitan region of Natal (64.4%) and that found in women from Pelotas [17], South Brazil (68.9%), but below the rates reported in women from São Paulo, Southeastern Brazil [18] (77.3%). It was also found that 18.0% of the respondents had not been tested with the recommended frequency, and 12.6% reported that they had never been tested. These values are smaller when compared with those found in women from a municipality in the metropolitan region of Natal [16], whose percentages were 35.6% for those who had not been tested with the recommended frequency and 15.0% for those who had never been tested. The proportion of women who had never had a Pap test in their life, as found by this study, is similar to that found in São Paulo [18] (13.9%) and in women of Campinas [8] (11.2%), both located in southeastern Brazil, but is well above that found in women of Medellin, Colombia [7] (7.8%).

The results show that the surveyed women had a very low knowledge level about the screening test for cervical cancer, since only 47.0% had a medium or high level of knowledge about the exam, compared with the women from Medellin [7], where this rate reached a value of 97.3%. Our study found a greater proportion of women with a high level of knowledge about the Pap test among those with more education and higher household income. This is probably due to more ease of access available to these women for information on the examination. Our results are concordant in this aspect with those reported for women of Medellin [7]. Also, an association was observed between the middle level of knowledge about the Pap test with marital status and pregnancy, where women married or living in a stable relationship with her partner and those who had at least one pregnancy presented a higher degree of knowledge about the exam. This could be explained by the fact that these women more frequently seek health services for guidance on contraceptive methods or for prenatal exams.

Regarding the knowledge of the women from this study about HPV, although it was very low with only 29.1% of those interviewed showing a medium or high degree of knowledge on the virus, this proportion is above that described for the women of Medellin [7], but far below that found in women of Sweden [19]. In two other studies conducted in Brazil, involving public school students, one study being performed in the South region [20] and another in the Southeast region [21], very low levels of knowledge about HPV were also found. We observed a greater proportion of women with high-level knowledge about HPV among those aged over 38 years, in those with greater schooling, among the married women or those living in a stable relationship with their partner, in those with higher household incomes, and in those who had had at least one pregnancy. The reason may be that these women have greater ease of access to information on HPV and its role in the development of premalignant and malignant lesions of the genital tract and possibly that these women more frequently seek health services for guidance about contraceptive methods or for prenatal exams. These results are similar to those obtained for women from Medellin [7], regarding the variables of age, education, and socio-economic condition, but differ with respect to marital status. With respect to the association of the high level of knowledge on HPV and the variables marital status and education, our results are also similar to those described for women of Quebec, Canada [22].

Although almost all participants said that they had heard about the Pap test, only a minority could talk about its purpose but, nonetheless, were unaware of the advantages and benefits of having the procedure regularly to prevent cervical cancer. However, a significant proportion (69.4%) of the respondents said they had undergone the examination with the frequency recommended by the Health Ministry. This suggests that most of the women had the examination because they were recommended to do so, but they did not know the purpose it served, nor did they possess any degree of awareness about the advantages and benefits of the procedure for their health. On the other hand, a significant portion of respondents had heard about the HPV, and a smaller proportion knew that it is transmitted through sexual contact; however, the majority of them were unable to establish any correlation between genital infection with this virus and the occurrence of cervical lesions, including cancer.

The Brazilian public health system consists of the SUS (Sistema Único de Saúde-Unified Health System), which aims to provide full health coverage to every citizen of Brazil. Actions directed to family planning are inserted within the SUS through the Family Health Program (PSF). However, due to its wide scope, this system has limitations, which sometimes may make access to the health service difficult. Moreover, there is a low degree of knowledge and awareness of the women in relation to HPV and the importance of a periodical Pap test, aimed at the early detection and treatment of cervical lesions associated with HPV and cervical cancer prevention. This is reflected in the low levels of coverage obtained by the cervical cancer screening program. In this context, the findings of this study may serve to arouse the attention of professionals working at PSF about the need to intensify educational activities, aiming to improve the level of knowledge and awareness about the role of HPV in the etiology of uterine cervical lesions, including cervical cancer, and the importance of performing periodic cytologic exams to prevent these diseases.

The present study has some limitations. First, the target population included only women studying in public schools or attending public health units, a population who generally present a lower socioeconomic status and who may not be so representative of the women population of Natal city. Secondly, questions about the HPV vaccine were not included in this research. Although the vaccine is the most effective way to prevent HPV infection, it is not accessible to the majority of the population, since it is not yet effectively included in the Brazilian official program of vaccination.

## 5. Conclusion

Our results show that the majority of women of the segment of the studied population have low levels of knowledge and of conscientization of the role of HPV as a causative agent of precancerous and cancerous lesions of the anogenital tract. Furthermore, the majority of women respondents do not have adequate knowledge about the screening test for cervical cancer or about the advantages and benefits of doing so regularly to prevent the disease. Thus, it is clear there is a critical need to improve the knowledge of the local female population on the role of HPV in the etiology of cervical cancer, as well as about the screening method of this disease. Therefore, we suggest educational campaigns emphasizing the role of HPV in the etiology of the cervical lesions of different degrees, including cancer, as well as the importance of regular Pap tests to prevent these diseases.

## Figures and Tables

**Table 1:** Demographic and behavioral characteristics of survey respondents in the metropolitan region of Natal, Brazil.

Característica	*N*	%
Age (years)		
14–28	366	51.8
29–38	180	25.5
39–48	92	13.0
49–59	68	9.6
Ethnicity		
White	337	47.7
Nonwhite	369	52.3
Education		
Elementary school or less	262	37.1
Attending high school or high school	370	52.4
Above high school	74	10.5
Marital status		
Single	253	35.8
Married or accompanied	453	64.2
Household income		
Low income	557	78.9
Middle or high income	121	17.1
High income	28	4.0
Pregnancy		
No	375	53.1
Yes	331	46.9
Number of pregnancies		
0	375	52.8
1–3	268	38.0
≥4	63	8.9

**Table 2:** Frequency, distribution, and univariate associations of characteristics in relation to Pap knowledge level.

Characteristic	*N* = 706	Pap test knowledge score			*P*
		Low	Medium	High	
Age (years)					0.237
14–28	366	207 (56.6)	78 (21.3)	81 (22.1)	
29–38	180	93 (51.7)	47 (26.1)	40 (22.2)	
39–48	92	39 (42.4)	29 (31.5)	24 (26.1)	
49–59	68	35 (51.5)	20 (29.4)	13 (19.1)	
Ethnicity					0.484
White	337	173 (51.3)	82 (24.3)	82 (24.3)	
Nonwhite	369	201 (54.5)	92 (24.9)	76 (20.6)	
Education					0.000
Elementary school or less	262	171 (65.3)	79 (30.2)	12 (4.6)	
High school and above	444	203 (45.7)	95 (21.4)	146 (32.9)	
Marital status					0.000
Single	253	157 (62.0)	37 (14.6)	59 (23.3)	
Married or accompanied	453	217 (47.9)	137 (30.2)	99 (21.8)	
Household income					0.000
Low income	557	317 (56.9)	138 (24.8)	102 (18.3)	
Middle or high income	149	57 (38.3)	36 (24.2)	56 (37.6)	
Pregnancy					0.000
No	375	230 (61.3)	60 (16.0)	85 (22.7)	
Yes	331	144 (43.5)	114 (34.4)	73 (22.1)	

**Table 3:** Frequency, distribution, and univariate associations of characteristics in relation to HPV knowledge level.

Characteristic	*N* = 706	HPV knowledge score			*P*
		Low	Medium	High	
Age (years)					0.000
14–28	366	260 (71.0)	86 (23.5)	20 (5.5)	
29–38	180	125 (69.4)	42 (23.3)	13 (7.2)	
39–48	92	62 (67.4)	11 (12.0)	19 (20.7)	
49–59	68	54 (79.4)	2 (2.9)	12 (17.6)	
Ethnicity					0.061
White	337	225 (66.8)	78 (23.1)	34 (10.1)	
Nonwhite	369	276 (74.8)	63 (17.1)	30 (8.1)	
Education					0.000
Elementary school or less	262	237 (90.5)	24 (9.2)	1 (0.4)	
High school or above	444	264 (59.5)	117 (26.4)	63 (14.2)	
Marital status					0.006
Single	253	174 (68.8)	64 (25.3)	15 (5.9)	
Married or accompanied	453	327 (72.2)	77 (17.0)	49 (10.8)	
Household income					0.000
Low income	557	424 (76.1)	96 (17.2)	37 (6.6)	
Middle or high income	149	77 (51.7)	45 (30.2)	27 (18.1)	
Pregnancy					0.000
No	375	263 (70.1)	96 (25.6)	16 (4.3)	
Yes	331	238 (71.9)	45 (13.6)	48 (14.5)	

**Table 4:** Distribution of respondents by Pap knowledge level according to the considered variables and associated odds ratio (OR) obtained by univariate logistic regression model.

Characteristic	*N* = 706	Pap knowledge score				*P*
		Low or medium	High	OR	CI	
Education						0.000
Elementary school or less	262	250	12 (4.6)	1.00	[Reference]	
High school or above	444	298	146 (32.9)	10.20	[5.534–8.825]	
Household income						0.000
Low income	557	455	102 (18.3)	1.00	[Reference]	
Middle or high income	149	93	56 (37.6)	2.68	[1.810–3.987]	
Marital status						0.920
Single	253	194	59 (23.3)	1.00	[Reference]	
Married or accompanied	453	354	99 (21.8)	0.92	[0.637–1.327]
Pregnancy						0.965
No	375	290	85 (22.7)	1.00	[Reference]	
Yes	331	258	73 (22.1)	0.97	[0.677–1.377]	

**Table 5:** Distribution of respondents by HPV knowledge level according to the considered variables and associated odds ratio (OR) obtained by univariate logistic regression model.

Characteristic	Number of percentage of women (%) HPV knowledge score					*P*
	*N* = 706	Low or medium	High	OR	CI	
Age (years)						
14–28	366	346	20 (5.5)	1.00	[Reference]	
29–38	180	167	13 (7.2)	1.35	[0.654–2.773]	0.419
39–48	92	73	19 (20.6)	4.50	[2.289–8.859]	0.000
49–59	68	56	12 (17.6)	3.71	[1.718–8.001]	0.001
Education						
Elementary school or less	262	261	1 (0.4)	1.00	[Reference]	
High school or above	444	381	63 (14.2)	43.16	[5.948–3.130]	0.000
Marital status						
Single	253	238	15 (5.9)	1.00	[Reference]	
Married or accompanied	453	404	49 (10.8)	1.92	[1.056–3.507]	0.033
Household income						
Low income	557	520	37 (6.6)	1.00	[Reference]	
Middle or high income	149	122	27 (18.1)	3.11	[1.824–5.305]	0.000
Pregnancy						
No	375	359	16 (2.3)	1.00	[Reference]	
Yes	331	283	48 (14.5)	3.81	[2.116–6.844]	0.000
